# Supplementary material for: Detecting quadrupole: a hidden source of magnetic anisotropy for Manganese alloys
Source: Sci Rep. 2020 Jun 16;10:9744. doi: 10.1038/s41598-020-66432-9 (PMC7297735; doi:10.1038/s41598-020-66432-9)
Supplement: Supplementary file 1 — Supplementary Information. [file 41598_2020_66432_MOESM1_ESM.pdf]

Supplemental Materials (Note and Figures S1-S4)

Detecting quadrupole: a hidden source of magnetic anisotropy for Manganese alloys

Jun Okabayashi[1], Yoshio Miura[2], Yohei Kota[3],

Kazuya Z. Suzuki[4,5], Akimasa Sakuma[6,5,7], and Shigemi Mizukami[4,5,7]

[1]*Research Center for Spectrochemistry, The University of Tokyo, 113-0033 Tokyo, Japan*

[2]*Research Center for Magnetic and Spintronic Materials, National Institute for Materials Science (NIMS), Tsukuba 305-0047, Japan*

[3]*Fukushima College, National Institute of Technology, Iwaki, Fukushima,970-8034, Japan*

[4]*WPI-Advanced Institute for Materials Research, Tohoku University, Sendai 980-8577, Japan*

[5]*Center for Spintronics Research Network (CSRN), Tohoku University, Sendai 980-8579, Japan*

[6]*Department of Applied Physics, Tohoku University, Sendai 980-8579, Japan and*

[7]*Center for Science and Innovation in Spintronics (CSIS), Tohoku University, Sendai 980-8577, Japan*

SUPPLEMENTAL NOTE

Estimation of spin and orbital magnetic moments from XAS and XMCD spectra

Magneto-optical sum rules for XMCD state that the transition intensity is proportional to the valence hole number n_h and described by the following relations;

$$m_{\text{orb}} = \frac{4}{3}\left[\frac{\Delta I_{L3} + \Delta I_{L2}}{I_{L3} + I_{L2}}\right] n_h \tag{1}$$

$$m_{\text{spin}} - 7m_{\text{T}} = 2\left[\frac{\Delta I_{L3} - 2\Delta I_{L2}}{I_{L3} + I_{L2}}\right] n_h, \tag{2}$$

using the integrals of XAS spectra after subtracting the integral background, the values of $I_{L3} + I_{L2}$ are estimated. XMCD intensities are written as ΔI_{L3} and ΔI_{L2}. Using the integrals of XMCD of $\Delta I_{L3} + \Delta I_{L2}$, m_{orb} can be estimated. The m_{spin} values including magnetic dipole term m_{T} are calculated by $\Delta I_{L3} - 2\Delta I_{L2}$.

A conventional application of sum rules were developed by C.T. Chen *et al.* (Phys. Rev. Lett. **75**, 152 (1995)), where using the values of $I_{L3} + I_{L2}$, $\Delta I_{L3} + \Delta I_{L2}$, and $\Delta I_{L3} - 2\Delta I_{L2}$ as r, q, and $4p - 6q$, respectively. m_{orb} and m_{spin} are described as $4q/3r\ n_h$ and $6p - 4q/r\ n_h$, respectively. The p, q, and r values are estimated from the convergent. The values n_h are determined from the first-principles calculation. Since the estimation of $I_{L3} + I_{L2}$ includes the ambiguities through the estimation of back ground contributions, the error bars are estimated to be ± 20 % for m_{orb} and m_{spin}.

Estimation of quadrupole tensor Q_{zz} from XAS and XMLD spectra

Magneto-optical sum rule for XMLD states that the transition intensity is proportional to the valence hole number n_h and described by the following relations;

$$Q_{zz} = \frac{l(2l-1)(4l+2-(10-n_h))}{2}\left[\frac{\Delta I_{L3} + \Delta I_{L2}}{I_{L3} + I_{L2}}\right] \tag{3}$$

using the integrals of XAS spectra after subtracting the integral background, the values of $I_{L3} + I_{L2}$ are estimated. Here, an angular momentum number of valence $3d$ electrons $l = 2$ is adopted. XMLD intensities are written as ΔI_{L3} and ΔI_{L2}. Using the integrals of XMLD; $\Delta I_{L3} + \Delta I_{L2}$, Q_{zz} can be estimated using the notation described in P. Carra *et al.*,

Physica B **192**, 182 (1993). For $3d$ transition mental compounds, positive convergent values of XMLD integrals directly rink to the positive sign of Q_{zz} in this formulation.

SUPPLEMENTAL INFORMATION

In this supplemental information, we add short explanation about Figs. S1-S4.

Figure S1 shows the Ga L-edge XAS and XMCD of Mn_1Ga taken at room temperature. Clear L_3 and L_2 edges are observed at around 1,112 and 1,138 eV, respectively. Corresponding induced XMCD signals are also shown. The sign of induced XMCD is the same as MnI site shown in Fig. 1 in the main text. Further, there are satellite structures at around 1,118 and 1,125 eV for L_3 edge. These originate from one of the extended x-ray absorption fine structure oscillations. For these peaks, there are no XMCD intensities.

Figure S2 displays the bar graph of the atomic contributions for magnetic anisotropy energy (MAE) of L_x and L_z matrix elements in $L1_0$-Mn_1Ga. The lattice constant of $a = 2.75$ Å and c/a =1.32 is adopted, which is determined by the x-ray diffraction experiment. The second-order perturbation terms for spin-orbit interacting in d orbitals are listed with spin-conserved and spin-flipped cases. Each matrix element $E^{i,j}_{z(x)}$ is written as follow.

$$E^{i,j}_{z(x)} = \xi^2 \sum_{u,o} \frac{|\langle o, d(i)|L_z(x)|u, d(j)\rangle|^2}{\epsilon_{k,u} - \epsilon_{k,o}}$$

Positive MAE stabilizes the PMA. Since the positive and negative contributions are canceled out in each matrix element, the residuals contribute to the MAE.

Figure S3 also displays the similar case with Fig. S2 for $D0_{22}$-Mn_3Ga decomposing into MnI and MnII sites. The lattice constant of a =3.898 Å and c/a =1.8156 is adopted.

Figure S4 shows the valence electron dependence in MAE deduced by the second-order perturbation of spin-orbit interaction. MAE consists of four contributions $\Delta E_{\rm up} + \Delta E_{\rm down} + \Delta E_{\rm T} + \Delta E_{\rm LS}$ as shown in Fig. S4, which exhibits that the contribution of $E_{\rm T}$ is dominant to describing electron number dependence. The position of N =10 exhibits the E_F and the contributions at E_F for each component is plotted in the bar graph. $\Delta E_{\rm T}$ contributes strongly to stabilize the PMA.

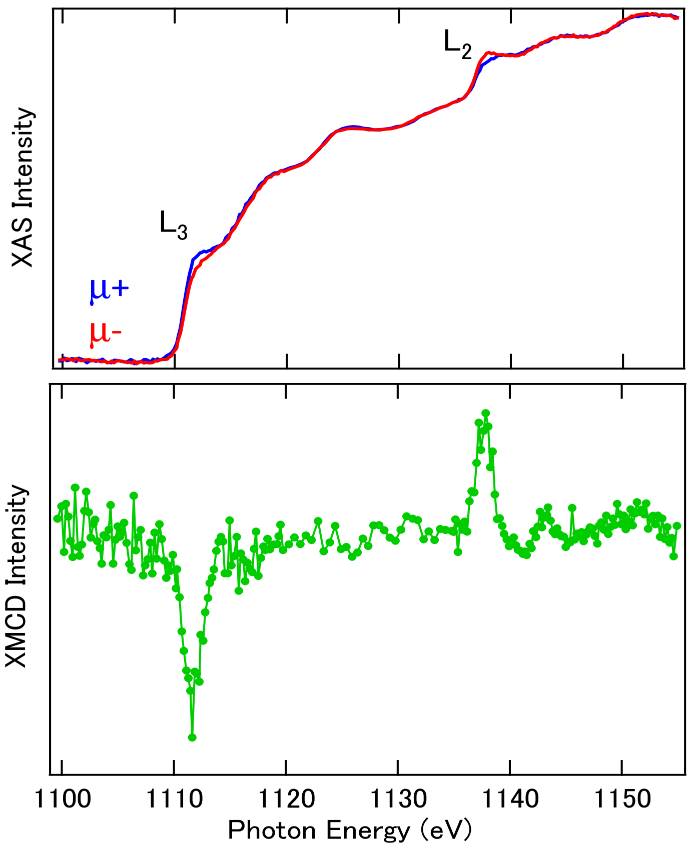

Fig. S1, XAS and XMCD in Ga L-edges of Mn_1Ga.

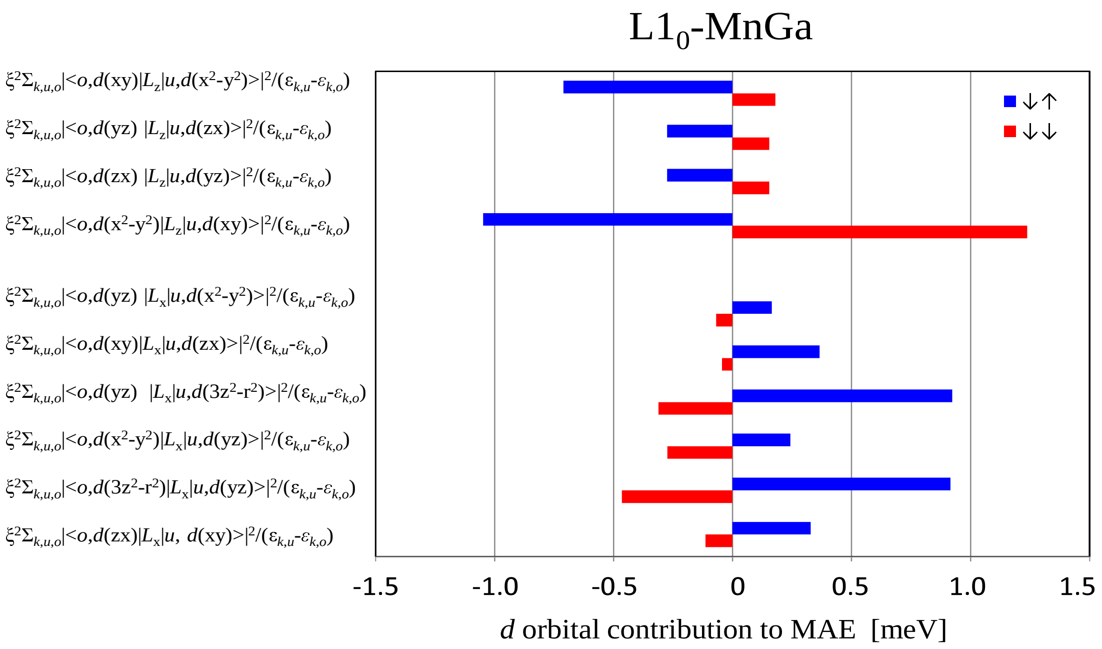

Fig. S2, Contributions of orbital angular momenta matrix elements to MAE for unforbidden transitions in Mn_1Ga. Red and blue bars are spin-conserved and spin-flipped transitions, respectively.

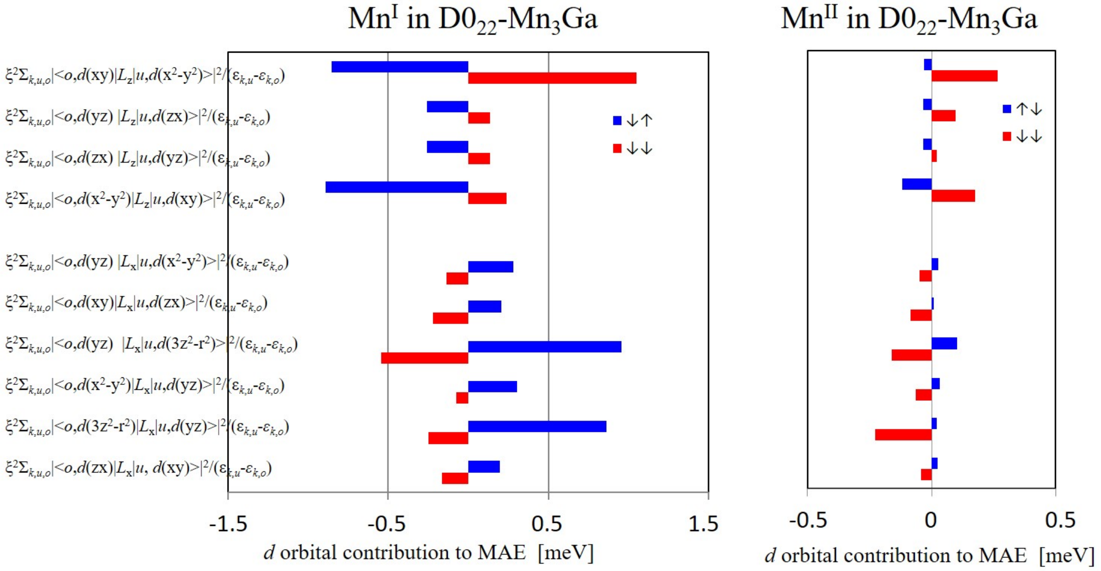

Fig. S3, Contributions of orbital angular momenta matrix elements to MAE for unforbidden transitions in Mn_3Ga. Red and blue bars are spin-conserved and spin-flipped transitions, respectively.

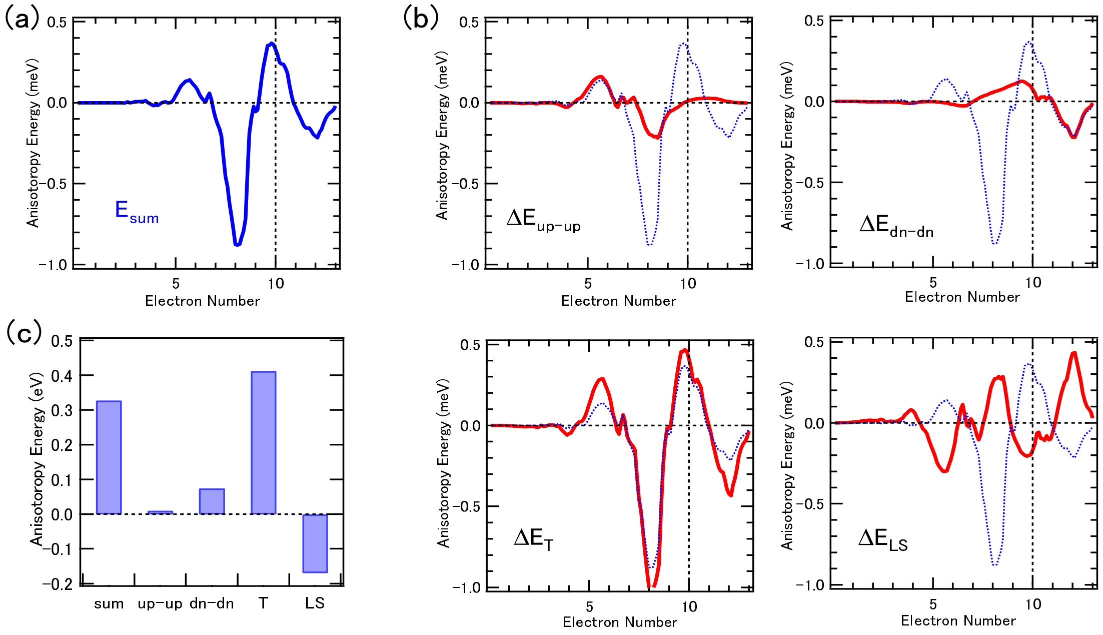

Fig. S4, MAE in Mn_1Ga as a function of valence electrons, calculated by the perturbation of the spin-orbit coupling energy. (a) Sum of each component; $\Delta E_{\rm up}$, $\Delta E_{\rm down}$, $\Delta E_{\rm T}$, and $\Delta E_{\rm LS}$ terms (solid-red-lines). (b) Decomposed components. Dot-blue-lines are eye guide in (a). (c) Bar graph of the contribution of each component at the E_F, where electron number N=10.
